# Short persistence of bendiocarb sprayed on pervious walls and its implication for the indoor residual spray program in Ethiopia

**DOI:** 10.1186/s13071-016-1549-7

**Published:** 2016-05-05

**Authors:** Yemane Yeebiyo, Dereje Dengela, Alemayehu Getachew Tesfaye, Gedeon Yohannes Anshebo, Lena Kolyada, Robert Wirtz, Sheleme Chibsa, Christen Fornadel, Kristen George, Allison Belemvire, Hiwot Solomon Taffese, Bradford Lucas

**Affiliations:** The President’s Malaria Initiative Africa Indoor Residual Spraying Project, Abt Associates, Gerji Road, Sami Building, 1st Floor, Addis Ababa, Ethiopia; The President’s Malaria Initiative Africa Indoor Residual Spraying Project, Abt Associates, 4550 Montgomery Ave., Suite 800 North, Bethesda, MD 20814 USA; Entomology Branch, Centers for Disease Control and Prevention, 1600 Clifton Road, Atlanta, GA 30329-4027 USA; U.S. Agency for International Development (USAID), Entoto Street, Addis Ababa, Ethiopia; President’s Malaria Initiative, United States Agency for International Development, Bureau for Global Health, Office of Health, Infectious Disease & Nutrition, 10082A, 2100 Crystal Drive, Arlington, VA 22202 USA; National Malaria Control Program, Federal Ministry of Health, Addis Ababa, Ethiopia

**Keywords:** IRS, Bendiocarb, Propoxur, *Anopheles arabiensis*, Vector control, Experimental hut, Ethiopia

## Abstract

**Background:**

With the emergence and spread of vector resistance to pyrethroids and DDT in Africa, several countries have recently switched or are considering switching to carbamates and/or organophosphates for indoor residual spraying (IRS). However, data collected on the residual life of bendiocarb used for IRS in some areas indicate shorter than expected bio-efficacy. This study evaluated the effect of pH and wall type on the residual life of the carbamates bendiocarb and propoxur as measured by the standard World Health Organization (WHO) cone bioassay test.

**Methods:**

In phase I of this study, bendiocarb and propoxur were mixed with buffered low pH (pH 4.3) local water and non-buffered high pH (pH 8.0) local water and sprayed on two types of wall surface, mud and dung, in experimental huts. In the six month phase II study, the two insecticides were mixed with high pH local water and sprayed on four different surfaces: painted, dung, mud and mud pre-wetted with water. The residual bio-efficacy of the insecticides was assessed monthly using standard WHO cone bioassay tests.

**Results:**

In phase I, bendiocarb mixed with high pH water killed more than 80 % of susceptible *Anopheles arabiensis* mosquitoes for two months on both dung and mud surfaces. On dung surfaces, the 80 % mortality threshold was achieved for three months when the bendiocarb was mixed with low pH water and four months when it was mixed with high pH water. Propoxur lasted longer than bendiocarb on dung surfaces, staying above the 80 % mortality threshold for four and five months when mixed with high and low pH water, respectively. Phase II results also showed that the type of surface sprayed has a significant impact on the bio-efficacy of bendiocarb. Keeping the spray water constant at the same high pH of 8.0, bendiocarb killed 100 % of exposed mosquitoes on impervious painted surfaces for the six months of the study period compared with less than one month on mud surfaces.

**Conclusions:**

Mixing the insecticides in alkaline water did not reduce the residual bio-efficacy of bendiocarb. However, bendiocarb performed much better on impervious (painted) surfaces than on porous dung or mud ones. Propoxur was less affected by wall type than was bendiocarb. Studies on the interaction between wall materials, soil, humidity, temperature and pH and the residual bio-efficacy of new and existing insecticides are recommended prior to their wide use in IRS.

## Background

Indoor residual spraying (IRS) of houses with insecticide has been used for over 70 years to reduce malaria transmission by killing and/or repelling mosquitoes that transmit malaria parasites [1]. IRS with dichlorodiphenyltrichloroethane (DDT) dates back to 1944 [2]. In the early years, this approach significantly reduced the vector population and malaria burden [3]. By the 1950s, the results produced such optimism among malaria endemic countries, malaria control communities, donors and multilateral organizations that a World Health Organization (WHO)-led global malaria eradication campaign was launched in 1955 [2–5]. While the program eliminated malaria in countries in Europe and North America, as well as in the former Soviet Union and most Caribbean islands [2], it was not implemented in all malaria endemic countries and thus did not achieve its goal of eradicating malaria worldwide.

Malaria eradication pilot projects that implemented IRS with DDT in several African countries proved to be effective in reducing vector density and longevity as well as cases of malaria including in areas with high malaria transmission, but they were unable to interrupt malaria transmission [3–5]. This shortcoming, along with the lack of road and communication systems needed to do IRS, discouraged malaria policy makers and led to exclusion of a large part of the continent from the eradication campaign [4, 5]. As a result, only three countries in Africa, including Ethiopia, were selected to upgrade their pilot programs into a national eradication campaign [3]. Ethiopia launched a malaria eradication program in 1959 based on IRS with DDT [6]. The time-limited program was ultimately deemed unsuccessful for reasons that included the absence of a similar initiative in neighboring countries, lack of basic infrastructure, and a global shift in goal from eradication to more limited control dictated by a decline in financial resources [6, 7]. In 1971, Ethiopia switched from eradication to control, modifying its IRS strategy from one of blanket coverage of all malaria endemic areas to selective application in high burden and epidemic prone locations [6]. IRS has continued to play a major role in malaria vector control in Ethiopia. DDT was the insecticide of choice until 2009, when data began to show high levels of vector resistance to DDT and pyrethroids [8–12]. Since then, IRS programming in Ethiopia has been guided by data on local vector resistance to insecticides recommended for IRS by the WHO Pesticide Evaluation Scheme (WHOPES) and the residual efficacy of the insecticides.

Recently, the Ethiopian IRS program began to use two insecticides in the carbamate class, bendiocarb and propoxur. However, there are concerns about the residual life of both insecticides because they are known to have shorter periods of residual efficacy than DDT and some pyrethroids [13]. Their chemical composition and formulation can interact with external factors, such as the water used to mix the insecticides and the type of wall material to which they are applied, affecting their residual life on sprayed walls. Several unpublished reports indicate that carbamates can undergo alkaline hydrolysis when the water used to mix the insecticides has a pH higher than 7 [14]. Some carbamates also degrade more quickly in solutions containing iron and copper ions [15]. A recent study in Tanzania reported that pH has a significant influence on the residual efficacy of lambda-cyhalothrin (ICON 10CS). In that study, lambda-cyhalothrin residual efficacy on walls of mud daub, limestone blocks, cement blocks, whitewash and oil painted surfaces with a pH ranging from 10.2 to 11.5 was below the threshold of ≥ 80 % mortality at 42–56 days after spraying. Walls made of iron sheets, palm thatch (pH = 5.95) and wood (pH = 7.3) retained the recommended efficacy of ≥ 80 % mortality beyond 152 days [16].

Studies assessing the effect of different types of wall surfaces on the decay rate of carbamates have reported mixed results. A study in Mozambique found that bendiocarb had an effective residual life of six months; different types of wall surfaces did not affect the decay rate [17]. In a study from Zimbabwe, the effective residual life of bendiocarb was longer on a thatch surface than on a mud surface [18]. Similarly, in trials in Cameroon, bendiocarb had a knock-down effect for longer periods on wood and cement surfaces than on mud walls [19]. Djenontin et al. found bendiocarb at a target dose of 400 mg/m^2^ kills at least 80 % of exposed mosquitoes for 13 weeks on teak wood, seven weeks on cement and six weeks on red clay and a mixture of red clay and cement [20]. A study in the Gambia found bendiocarb resulted in more than 80 % mortality for at least five months on mud walls. However, the dose of bendiocarb in this experiment was 980 mg/m^2^, more than double the recommended dose of 400 mg/m^2^ [21]. Another study found that incorporating carbamates into a paint binder or adsorbing them into phosphogypsum prolonged their effective lifespan [22].

The effect of surface porosity, sorption, soil type and climatic factors on the bio-efficacy of insecticides was the focus of several studies in the 1950s [23–28]. However, interest in such studies diminished as the aim of the global program shifted from eradication to control and many countries largely abandoned IRS as a vector control tool. But the past decade has seen renewed and significant interest as the Global Fund to Fight Aids, Tuberculosis and Malaria and the President’s Malaria Initiative (PMI) have scaled up their support of IRS in African countries, making such studies relevant and timely.

This study was conducted to guide the PMI IRS operation and the Ethiopia national IRS program in selecting an appropriate insecticide for use in light of the high level of resistance that vectors have developed to DDT and pyrethroids. No studies on the residual efficacy of carbamates have taken into account how the lifespan of these chemicals is affected by the composition of water used to mix the chemicals, or the different types of wall surfaces found in Ethiopia. Communities in rural areas use different building materials to build their houses depending on local culture and traditions, soil type, climate, lifestyle and wealth. Few people in rural Ethiopia can afford to build houses with cemented or painted walls. More common are houses with porous mud surfaces or mud walls smoothed with dung or limestone, and the mud walls themselves vary by location due to the content of the soil and the process used to make the mud plaster. This study focused on assessing the role of wall types and water pH on the effective residual life of the carbamate insecticides bendiocarb and propoxur.

## Methods

This study was conducted in two phases in a semi-arid environment in Adulala village about 5 km from the town of Nazareth and 95 km southeast of Addis Ababa, the capital city of Ethiopia. The study area is known for unstable and seasonal transmission of malaria. Phase I assessed the effect of pH on the residual bio-efficacy of carbamates; phase II evaluated the residual life of carbamates sprayed on different wall surfaces.

### Construction of experimental huts

Twelve circular experimental huts were constructed for this study. The 12 experimental huts were identical in size and design and represented a typical house of the local community. The circular huts were 3 m in diameter, had approximately 2.5 m-high walls, and had a cone-shaped, thatched roof, the most common roofing material in Ethiopia. They also had a 50 × 50 cm wooden window and a 2 m × 90 cm wooden door. The walls of the huts were made of wood plastered with several layers of mud according to local practice. In phase I, the mud walls of 6 of the houses were coated or smoothed with cattle dung; the other 6 were left with a mud surface. In phase II, 4 huts were dung coated, 4 were treated with acrylic paint to create a water resistant surface and 8 were left with mud surfaces wetted with water or dry. All huts were locked throughout the study period except when the wall assays were performed.

### Mixing and insecticide spraying

The walls of the huts were sprayed with bendiocarb (FICAM 80 % WP, Bayer) and propoxur (50 % WP, produced by the Adami Tulu pesticide processing plant) at 0.4 g and 2 g active ingredient respectively per square meter surface area. The bendiocarb came from USAID/PMI, and had been procured by the USAID Ethiopia country office from Bayer for use in the PMI IRS program. The propoxur was obtained from the Federal Ministry of Health of Ethiopia, which procured the insecticide locally from Adami Tulu. The insecticides were fresh batches, less than six-months-old after production, kept under ideal storage conditions (18–24 degree Celsius) prior to their use in this study. The suppliers of both insecticides presented certificates of analysis from independent laboratories, which was a requirement for their product to be considered for purchase. The certificates indicated that these insecticides met all the specification set by WHOPES.

In phase I, the bendiocarb and propoxur were mixed in high pH (pH 8.0) local water and buffered low pH (pH 4.3) local water and sprayed on two types of wall surfaces, mud and dung. The pH of the water was lowered using the buffering agent Probuff®700. The study then evaluated the effective residual life of the two insecticides to see if the pH of the water used to mix the insecticides or the type of wall surface affected the residual life.

A Hudson X*-*Pert*®* pump with a flat nozzle (SS 8002) and discharge rate of 760 ml per minute was used to apply the insecticides on the walls. To maintain uniformity, the pressure gauge reader was kept at 35–55 lb per square inch (psi). To ensure the right amount of insecticide was uniformly applied to the walls of the experimental huts, a well-experienced and skilled spray operator who has served the program for more than 10 years was selected. Before spraying the experimental huts, his performance was tested and proven accurate on a spray operators’ training wall using water. In addition, the spraying process was strictly supervised by senior operation experts from the project.

#### Phase I: Tests using different pH and wall type conditions

The first phase of the study took place from April through October 2012. Data on insecticide residual life were collected in April, 24 h after the walls of the experimental huts were sprayed, and again monthly for 5 months, May through October. As noted above, the walls of 6 of the 12 huts were plastered with mud and the walls of the other 6 huts were coated with dung. Four of each group of huts were sprayed with insecticide (2 with bendiocarb and 2 with propoxur) mixed either with local high pH water or local water acidified using a buffering agent. Two control houses from each group were sprayed with either non-buffered or buffered water only (Table 1).

**Table 1:** Experimental hut treatment for the study on the effect of pH on decay rate of carbamates - Phase I

Wall surface	Hut no	Sprayed with:
Dung	1	Non buffered local water/High pH (8.0)
	2	Buffered water/Low pH (4.3)
	3	Bendiocarb (0.4 g/m^2^) in High pH spray water
	4	Bendiocarb (0.4 g/m^2^) in Low pH spray water
	5	Propoxur (2 g/ m^2^) in High pH spray water
	6	Propoxur (2 g/ m^2^) in Low pH spray water
Mud	7	Non buffered local water/High pH (8.0)
	8	Buffered water/Low pH (4.3)
	9	Bendiocarb (0.4 g/m^2^) in High pH spray water
	10	Bendiocarb (0.4 g/m^2^) in Low pH spray water
	11	Propoxur (2 g/ m^2^) in High pH spray water
	12	Propoxur (2 g/ m^2^) in Low pH spray water

To determine whether any difference in residual efficacy was associated to the pH of the sprayed surfaces, the pH of the dung and mud surfaces of the experimental huts was also assessed using a battery-operated, handheld digital pH meter (model pHTestr®10 by Eutech Instruments) at the end of the study. To do this, the top surfaces of the mud and dung were carefully removed and mixed with distilled water (pH = 7) in a weight by volume ratio of 1 g sieved dust to 5 ml distilled water. The mixture was shaken and pH measures were taken.

#### Phase II: Tests on different wall types of varying porosity

As a follow up to the preliminary findings of the first trial, the second phase of the study assessed the lifespan of the two carbamates sprayed on four types of wall surfaces (Table 2). In addition to the (dry) mud and dung walls of phase I, phase II tested painted walls and mud walls wetted with local water (of the same pH as the mixing water). Painted walls were tested to see if their impervious surfaces extended the bio-availability of the carbamates for longer periods. Mud walls in 4 huts were wetted immediately before insecticides were sprayed to see if water temporarily closed the pores in the surface, potentially resulting in increased insecticide bio-availability by slowing down the sorption process.

**Table 2:** Experimental hut treatment for the study on the effect of wall surface on the decay rate of carbamates - Phase II

Wall surface	Hut no.	Sprayed with:
Dung	1	Propoxur (2 g/ m^2^)
	2	Bendiocarb (0.4 g/m^2^)
	3	Local water only
Painted	4	Propoxur (2 g/ m^2^)
	5	Bendiocarb (0.4 g/m^2^)
	6	Local water only
Dry mud	7	Water then propoxur (2 g/ m^2^)
	8	Water then bendiocarb (0.4 g/m^2^)
	9	Local water only
Wetted mud	10	Propoxur (2 g/ m^2^)
	11	Bendiocarb (0.4 g/m^2^)
	12	Local water only

Therefore, 3 experimental huts were treated with acrylic based paint and allowed to dry for 2 weeks. Of the remaining nine huts, 3 each had the following surfaces: dung plastered, dry mud and mud walls wetted with local water. One hut from each group was sprayed with bendiocarb, one with propoxur and one with local water only. As in the first phase of the study, mortality was assessed at 24 h after spraying, and then monthly for six months.

#### Cone wall bioassay

WHO cone bioassays [29] were used to monitor the durability of bendiocarb and propoxur on the sprayed walls. The study used known susceptible colonies of *Anopheles arabiensis* (Adama strain) reared at the Nazareth insectary in Ethiopia. Two-to-three-day-old female mosquitoes fed with sugar were used for the test. Ten to fifteen females were transferred into paper cups covered with netting. The mosquitoes were taken to the field for the test in a box covered with a moist towel to maintain humidity. In each hut, three cones were fixed to interior walls at 0.5 m, 1.0 m and 1.5 m above the ground to evaluate the residual bio-efficacy of the insecticides at different heights. A fourth cone was used as a control and was fixed on an insecticide-free layer of plain paper attached to the wall. Then, mosquitoes from each paper cup were transferred into the cones using a mouth aspirator. After 30 min of exposure, the mosquitoes were returned to the paper cups, which were kept in a wooden box covered with a moist towel. The mosquitoes were provided 10 % glucose solution during the 24 h holding period. Mortality was counted after 24 h. A mosquito was considered alive if it was able to fly.

The study had two sets of controls: (i) each experimental hut tested had a separate cone with mosquitoes exposed to unsprayed surface and (ii) experimental huts sprayed with local water or water with buffering agent only were also used as controls to exclude any effects from the local water and buffering agent on mortality.

#### Data analysis

When control mortality was between 5 and 20 % experimental mortality was corrected using Abbott’s formula [30]. The outcome variable was the number of mosquitoes dead after 30 min of exposure to sprayed walls and a further 24 h observation period. Mortality was calculated as the proportion of dead mosquitoes against the total number of mosquitoes exposed to each treatment.

Where appropriate, the Open Source Epidemiologic Statistics for Public Health (OpenEpi) version 3.03a program was used to calculate Chi-squared values and compare mortality rates between different treatments. We used negative binomial regression to test the statistical significance of differences in mosquito mortality exposed to two carbamate insecticides mixed in high and low pH water and sprayed on mud and dung plastered surfaces. A *P*-value of < 0.05 was considered significant. Bonferroni correction was used to ensure that the overall significance level did not exceed alpha, which was 0.05, when multiple comparisons were made. Stata 12 was used to run the statistical analysis.

Corrected exposure mortality rates are reported throughout the paper. Mortality rates of mosquitoes exposed to walls treated with control substances - either water or water with a buffering agent - were less than 5 % and were excluded from the analysis.

## Results

### Phase 1: Residual bio-efficacy of two carbamates mixed in high and low pH water and sprayed on dung and mud surfaces

#### High *vs* low pH of spray water

**Fig. 1:**
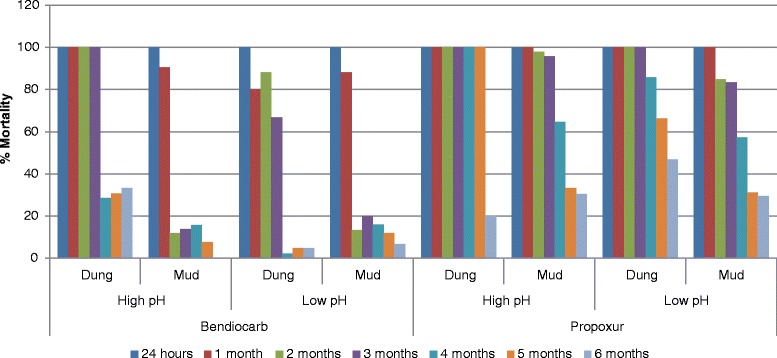
Mortality rate of susceptible mosquitoes exposed to different surfaces sprayed with bendiocarb and propoxur mixed in high and low pH water. Bendiocarb and propoxur are sprayed on mud or dung walls and mixed with either low or high pH water. Results of a six months wall bioassay test are presented. Dung walls performed better than mud walls and pH had no effect on residual efficacy

Bendiocarb mixed in high pH water and sprayed on dung walls killed more than 80 % of the exposed mosquitoes up to three months after spraying (Fig. 1). When bendiocarb mixed in high pH water was sprayed on mud surfaces, mosquito mortality of more than 80 % was achieved only for one month. Similar results were obtained when bendiocarb was mixed with low pH water and applied to dung surfaces, one month of bio-efficacy. Propoxur lasted for three months on mud surfaces irrespective of the pH of the water (high or low) that the insecticide was mixed with before spraying. On dung surfaces, it was effective for five months at high pH and slightly shorter (four months) using low pH water (Fig. 1). In a stratified analysis, which first stratifies by water pH and then by wall type and insecticide, a two-way comparison of the pH of the mixing water had no effect on the efficacy of either insecticide sprayed on mud surfaces. Mortality rates were 34.3 and 38.9 % (*χ*^*2*^ = 1.34*, P* = 0.24) for low and high pH water for bendiocarb, respectively, and 69.1 and 63.5 % (*χ*^*2*^ = 2.24*, P* = 0.13) for low and high pH water for propoxur, respectively (Table 3). On dung surfaces, both bendiocarb and propoxur performed better when mixed in high pH water as compared to when mixed in low pH water. For bendiocarb mortality rates were significantly higher (*χ*^*2*^ = 28.26, *P* < 0.001) on high pH than on low pH water; 71.6 % *vs* 50.6 %, respectively. The difference was also significant (*χ*^*2*^ = 34.51*, P* < 0.0001) for propoxur; 88.6 % for high pH *vs* 69.5 % for low pH mixing water (Table 3).

**Table 3:** The effect of wall surfaces and pH levels on performance of bendiocarb and propoxur

Insecticide	Low pH		High pH		Total by surface type		Total by pH level	
	Dung	Mud	Dung	Mud	Dung	Mud	Low pH	High pH
Bendiocarb	(156/308) 50.6^a^	(106/309) 34.3^b^	(219/306 ) 71.6^a^	(119/306 ) 38.9^b^	(375/614) 61.1^a^	(225/615) 36.6^b^	(262/617) 42.5^a^	(338/612) 55.2^b^
Propoxur	(219/315) 69.5^a^	(215/311) 69.1^a^	(279/315) 88.6^a^	(198/312) 63.5^b^	(498/630) 79.0^a^	(413/623) 66.3^b^	(434/626) 69.3^a^	(477/627) 76.1^b^
Total	(375/623) 60.2^a^	(321/620) 51.8^b^	(498/621) 80.2^a^	(317/618) 51.3^b^	(873/1244) 70.2^a^	(638/1238) 51.5^b^	(696/1243) 56.0^a^	(815/1239) 65.9^b^

Note: Change in letter across two columns connotes statistically significant difference of *P*(*χ* ^2^) < 0.0083. Numbers in the parenthesis are test results [number of mosquitoes dead (numerator) divided by total number of mosquitoes exposed (denominator)]

The pH of the surface scraped from the dung and mud walls was found to be 8.1 and 8.2, respectively, showing that there was no pH difference intrinsic to the two types of wall substrates.

#### Dung *vs* mud walls

Overall, the insecticides had significantly higher mosquito mortality on dung surfaces than on mud surfaces (*χ*^*2*^ = 90.54, *P* < 0.001), controlling for time, water pH, and insecticide (Table 3). For bendiocarb, effectiveness lasted longer on dung surfaces than on mud surfaces, killing 80 % or more mosquitoes for 2–3 months. Propoxur produced more than 80 % mosquito mortality for 4–5 months on dung surfaces and up to 3 months on mud surfaces, regardless of pH (Fig. 1).

#### Bendiocarb *vs* propoxur

Over the six month follow up period of phase I, bendiocarb killed 61.1 and 36.6 % of mosquitoes when sprayed on dung and mud surfaces, respectively (Table 3). The difference was statistically significant (*χ*^*2*^ = 73.75*, P* < 0.0001). Overall, propoxur killed 79.0 and 66.3 % of exposed mosquitoes on dung and mud surfaces, respectively. The difference between the two surface types was statistically significant (*χ*^*2*^ = 25.68, *P* < 0.0001). Propoxur performed better than bendiocarb (rate ratio = 0.557; CI: 0.311–0.803) on both mud and dung surfaces regardless of the water pH (Tables 3 and 4).

**Table 4:** Analysis of variation in mosquito mortality for two carbamates after controlling for pH of mixing water, type of wall and time

Parameter	Mortality rate ratio	*Z*	95 % CI	
			Min	Max
Wall type				
Mud	comparator	–	–	–
Dung	0.5961	3.4105**	0.2536	0.9387
Water pH				
High pH	comparator	–	–	
Low pH	-0.0475	-0.2689	-0.3934	0.2985
Insecticide				
Bendiocarb	comparator	–	–	
Propoxur	0.5570	4.4372*	0.3109	0.8030
Time				
24 h	comparator	–	–	–
Month 1	-0.0706	-0.3273	-0.4937	0.3524
Month 2	-0.5073	-2.2975*	-0.9401	-0.0745
Month 3	-0.4221	-1.9242	-0.8520	0.0079
Month 4	-1.5515	-6.4681**	-2.0216	-1.0813
Month 5	-1.2590	-5.4205**	-1.7142	-0.8038
Month 6	-1.2862	-5.5408**	-1.7411	-0.8312

*P < 0.05; **P < 0.01; *z* test statistic; *95 %CI* 95 confidence interval; *Min* minimum; *Max* maximum

### Phase II: Residual bio-efficacy of carbamates sprayed on surfaces of varying porosity

**Fig. 2:**
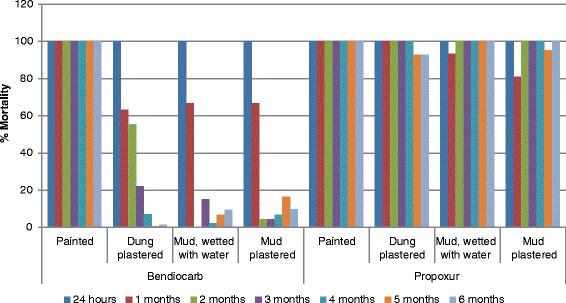
Mortality rate of mosquitoes exposed to different surfaces sprayed with bendiocarb and propoxur. Bendiocarb and propoxur are sprayed on dry mud, wetted mud, dung and pained walls. Results of a six months wall bioassay test are presented. The highest residual efficacy of bendiocarb was when sprayed on painted walls

In the second phase of the study, four types of surfaces were evaluated for their effect on the residual efficacy of carbamates. Propoxur demonstrated greater durability, with more than 80 % mortality throughout the six months of follow up irrespective of wall type (Fig. 2). Painted surfaces gave 100 % mortality up to six months after spray for both insecticides; for bendiocarb, this extended efficacy is highly significant compared with that of the other wall types (*P* < 0000.1) (Table 5). The mortality rate dropped below 80 % in less than a month after bendiocarb was sprayed on both mud surfaces, wetted and dry.

**Table 5:** Variation in mortality rate of mosquitoes exposed to different wall surfaces sprayed with bendiocarb and propoxur

Insecticide	Wall type	Exposed	Mortality (%)
Bendiocarb	Dung wall	282	41^a^
	Painted wall	281	100^b^
	Mud wall wetted with water	289	27^c^
	Mud wall	284	29^c^
Propoxur	Dung wall	301	99^b,d^
	Painted wall	279	100^b,d^
	Mud wall wetted with water	298	99^b,d^
	Mud wall	297	97^b,d^

Note: Change in letter connotes statistically significant difference of *P*(*χ* ^2^) < 0.0083

Dung surfaces performed significantly better than either dry mud surfaces (*χ*^*2*^ = 9.33*, P* = 0.002) or mud surfaces wetted with water (*χ*^*2*^ = 12.71*, P* = 0.0005) for bendiocarb, with about 10.0 % higher mortality. Dry and wetted mud walls resulted in 29 and 27 % mortality rates, respectively, which is not a statistically significant difference (*χ*^*2*^ = 0.25, *P* = 0.75).

Bendiocarb showed about a 50 % lower mortality than propoxur when averaged across time and type of wall surface (*χ*^*2*^ = 746.83*, P* < 0.0001). No difference of bio-efficacy for propoxur was detected across different types of walls.

## Discussion

The results of this study differ from previously published and unpublished reports that indicate that pH can affect the efficacy of carbamates and other insecticides as these chemicals can undergo alkaline hydrolysis in high pH spray water or surface. This study found no association between the pH of spray water and residual life and efficacy of carbamates sprayed on mud surfaces. The higher performance recorded on dung surfaces for both carbamates when mixed with high pH water as compared to mixing with low pH water was likely due to other factors unrelated to water pH, which need further investigation. Reports from elsewhere indicated that carbamates could be hydrolyzed in alkaline medium, potentially affecting their residual efficacies [14–16]. However, data from this study indicate that high pH water did not reduce the residual bio-efficacy of both carbamates when the carbamates are sprayed immediately after mixing, as was the case in this study. Time could be a factor in the process of hydrolysis - the insecticide may have to remain in a high pH water for some time for hydrolysis to take place and subsequently affect the residual bio-efficacy of the insecticide sprayed on the surface.

A study by Mutagahywa et al. in Tanzania showed that the pH of sprayed substrates impacted the residual life of the pyrethroid lambda-cyhalothrin [16]. The difference in residual life of the insecticides sprayed on mud and dung walls in our study cannot be attributed to an intrinsic difference in pH of the two surfaces because no pH difference was found. The reason for the difference in residual life of the insecticides might therefore be related to the porosity of the walls. Dung walls gave better results than mud ones. The mud surfaces used in this experiment were very rough and porous, like the walls most commonly found in houses in rural Ethiopia. Both laboratory and field studies have shown that insecticides perform better on non-porous surfaces than on porous ones [31], probably because the non-porous surface decreases the sorption rate and increases bio-availability.

The residual life of carbamates and organophosphates (another class of insecticides being considered as a replacement for DDT and pyrethroids in Ethiopia), has been reported to be strongly affected by atmospheric humidity and the sorption capacity of dried muds; thus the biological activity of a given concentration of insecticide increases with increasing humidity [32–36]. The investigators of this study did not measure the humidity of the environment during the test period. However, the field site where this study was undertaken is a semi-arid region with low humidity for most of the year, which may have contributed to lower residual efficacies on mud surfaces. Similarly, the lifespan of an insecticide is also influenced by interactions between the nature of the formulation of the insecticide and the type of the surface sprayed. Insecticides with high vapor pressure and volatility are more rapidly lost from non-sorbent surfaces such as glass and metal than insecticides with lower vapor pressures and less volatility [35]. Although no attempt was made here to determine the role of vaporization in the loss of the insecticides used, the carbamates tested in this experiment come in water dispersible powder formulations and loss due to volatility was likely not an issue.

The results of this study confirmed that less porous wall surfaces are effective in prolonging the bio-efficacy of bendiocarb on sprayed walls. The mortality rate remained at 100 % for six consecutive months on walls where paint almost completely closed the pores, reducing loss of insecticide from the surface. Wetting mud walls with water before applying insecticide, however, did not enhance bio-availability. This may be because wetting does not effectively close the pores in mud walls, and therefore does not reduce loss of insecticide from the surface. Moreover, wetted walls dried quickly, perhaps making the difference between wet and dry walls insignificant. Regarding dung plastered walls, results in the second phase of the study were similar to those in the first phase in that dung plastered surfaces gave better results than did wetted and dry mud surfaces, though the dung surfaces were inferior to painted ones.

Mud walls are most common in rural Ethiopia. Encouraging communities to use paints or local materials to smooth their walls might be incorporated into the health education program of the village health system in Ethiopia. However, the cost of painting houses could be beyond what most rural residents can afford. The literature shows that the relationship between porous surfaces and residual life of insecticides has been recognized since the 1950s and 1960s, and attempts have been made to inhibit sorption of organochlorines by modifying the formulation of the insecticide using products that can seal pores [37, 38]. Similar research on products that can reduce bendiocarb sorption by porous surfaces and prolong its residual life could potentially lower the decay rate of this chemical sprayed on mud walls.

The results presented here indicate that propoxur performed better than bendiocarb. In the second phase of the study, mean corrected mortality of mosquitoes exposed to propoxur and bendiocarb sprayed walls over the six months was 98.4 and 51.6 % respectively, a highly significant difference (*χ*^*2*^ = 746.83, *P* = 0.0001). The difference was consistent with buffered or non-buffered spray water and mud or dung walls. This deviates from the range of duration of effective action specified by WHO for both insecticides, which states 2–6 months for bendiocarb and 3–6 months for propoxur [13]. The volume of insecticide formulation (mixed in 8 l of water to make the insecticide suspension for spraying) was different for the two insecticides. While 800 g of 50 % propoxur formulation is added to 8 l of water to make the propoxur suspension and deposit 2 g active ingredient per meter square surface area, only 100 g of 80 % formulation is needed for bendiocarb for a dose of 0.4 g active ingredient per meter square, which is eight times less of the total volume and five times less of the active ingredient. The high volume of propoxur might have increased the viscosity of the propoxur suspension. However, further research would be needed to determine if viscosity contributed to reduced porosity of the walls, increasing the bioavailability of propoxur.

The IRS program in Ethiopia has few choices of insecticides for IRS due to resistance of the vector to DDT and almost all of the pyrethroids [8–12]. Therefore, making propoxur with its longer residual life more accessible on the global market is recommended as an option to increase the choice of insecticides for IRS. As a measure to address insecticide resistance and the short residual life of bendiocarb, it is highly likely that the IRS program in Ethiopia would shift to the more long-lasting formulation of organophosphate chemical, pirimiphos-methyl 300 CS, which is reported to have a longer residual life than bendiocarb [39–41]. However, assessment of its residual life that takes into account the different wall types common in rural Ethiopia and other factors is also recommended before its adoption for wide-scale use.

This study was designed to understand the impact of two specific factors, namely water pH and wall type, in the residual life of bendiocarb and propoxur, under experimental hut conditions and using a susceptible mosquito colony. The replicability of this result is unknown when using wild-caught, local mosquitoes in occupied houses where smoke from the cooking fire and other human activities might have some level of effect on the insecticide residual life. However, unpublished data collected by the PMI IRS program from occupied houses shows similar trends of residual bio-efficacy being affected by wall type for bendiocarb. To better understand the impact of these factors on the residual life of carbamates and learn their operational implications, it will be important to conduct further studies under natural conditions using residential houses and local, wild-caught mosquitoes.

This study has the limitation that no attempt was made to quantify the amount of insecticide deposited on the wall during the spray through the use of filter papers and chromatography or other methods. However, a spray operator with more than ten years of experience sprayed all the experimental huts to ensure that the right amount of the chemical was uniformly applied to the walls. The skill of the operator was tested before the spray and the process was strictly supervised by senior operational experts of the project. However, some variation in insecticide deposit cannot be fully excluded. Also the experimental huts were kept locked to avoid any human interference with the sprayed surfaces.

## Conclusion

This study provides evidence for use in choosing from among the limited options of insecticides currently available for IRS. Bendiocarb poorly fits spray operations where houses are built of porous soils, though it works well on painted and other non-porous surfaces. Propoxur seems to stay effective longer on all types of surfaces. The results of this study also suggest that the residual life of candidate insecticides for IRS needs to be critically assessed under local conditions before selection is made. The effect of different wall surfaces and pH on IRS residual efficacy is also scanty. Therefore, generalized recommendations on residual life of insecticides may not be enough to make decisions. Further assessment of local parameters, such as water/wall pH, wall surface type, soil, temperature, humidity and other factors on the effectiveness of IRS insecticides, is necessary to support evidence-based selections and timing of spray operations.

## Abbreviations

AIRS: Africa Indoor Residual Spraying
DDT: dichlorodiphenyltrichloroethane
IRS: indoor residual spraying
PMI: President’s Malaria Initiative
USAID: United States Agency for International Development
WP: wettable powder

## References

[1] World Health Organization (2014). World Malaria Report.

[2] Sadasivaiah S, Tozan Y, Breman JG (2007). Dichlorodiphenyltrichloroethane (DDT) for indoor residual spraying in Africa: how can it be used for malaria control?. Am J Trop Med Hyg.

[3] Mabaso LM, Sharp B, Lengeler C (2004). Historical review of malaria control in southern African with emphasis on the use of indoor residual house spraying. Trop Med Int Health.

[4] Alilio MS, Bygbjerg IC, Breman JG (2004). Are multilateral malaria research and control programs the most successful? Lessons from the past 100 years in Africa. Am J Trop Med Hyg.

[5] Trigg PI, Kondrachine AV (1998). Commentary. Malaria control in the 1990s. Bull World Health Organ.

[6] Oscar G (1992). Malaria eradication and the selective approach to health care: some lessons from Ethiopia. Int J Health Serv.

[7] Mekuria Y, Wolde TG (1970). Malaria survey in North and North Eastern Ethiopia. J Ethiopia Med.

[8] Yewhalaw D, Wassie F, Steurbaut W, Spanoghe P, Van Bortel W, Denis L (2011). Multiple insecticide resistance: an impediment to insecticide-based malaria vector control program. PLoS One.

[9] Balkew M, Gebre-Michael T, Hailu A (2003). Insecticide susceptibility level of *Anopheles arabiensis* in two agrodevelopment localities in eastern Ethiopia. Parassitologia.

[10] Yewhalaw D, Van Bortel W, Denis L, Coosemans M, Duchateau L (2010). First evidence of high knockdown resistance frequency in *Anopheles arabiensis* (Diptera: Culicidae) from Ethiopia. Am J Trop Med Hyg.

[11] Balkew M, Elhassen I, Ibrahim M, Gebre-Michael T, Engers H (2006). Very high DDT-resistant population of *Anopheles pharoensis* Theobald (Diptera: Culicidae) from Gorgora, Northern Ethiopia. Parasite.

[12] Fettene M, Olana D, Christian RN, Koekemoer LL, Coetzee M (2013). Insecticide resistance in *Anopheles arabiensis* from Ethiopia. Afr Entomol.

[13] World Health Organization. WHO recommended insecticides for indoor residual spraying against malaria vectors. 2009. http://apps.who.int/iris/bitstream/10665/46846/1/9789241564472_eng.pdf. Accessed 14 Jan 2014.

[14] McKie P, Johnson WS (2002). Water PH and its effect on pesticide stability.

[15] Strathmann TJ, Stone AT (2001). Reduction of the carbamate pesticides oxamyl and methomyl by dissolved Fe and Cu. Environ Sci Technol.

[16] Mutagahywa J, Ijumba JN, Pratap HB, Molteni F, Mugarula FE, Magesa SM (2015). The impact of different sprayable surfaces on the effectiveness of indoor residual spraying using a micro encapsulated formulation of lambda-cyhalothrin against *Anopheles gambiae* s.s. Parasit Vectors.

[17] Maharaj R, Casimiro S, Mthembu SD, Sharp BL (2004). The residual life of bendiocarb: a field-based evaluation from Mozambique. J Med Entom.

[18] Mpofu SM, Kanyimo KH, Masendu H (1991). Potential use of bendiocarb (Ficam VC) for malaria control in an area of Zimbabwe. J Am Mosq Cont Assoc.

[19] Etang J, Nwane P, Mbida JA, Piameu M, Manga B, Souop D (2011). Variations of insecticide residual bio-efficacy on different types of walls: results from a community-based trial in south Cameroon. Malar J.

[20] Djènontin A, Aïmihouè O, Sèzonlin M, Damien G, Ossè R, Soukou B (2013). The residual life of bendiocarb on different substrates under laboratory and field conditions in Benin, Western Africa. BMC Res Notes.

[21] Tangena JA, Adiamoh M, D’Alessandro U, Jarju L, Jawara M, Jeffries D (2013). Alternative treatments for indoor residual spraying for malaria control in a village with pyrethroid- and DDT-resistant vectors in The Gambia. PLoS One.

[22] Sibanda MM, Focke WW, Labuschagne FJWJ, Moyo L, Nhlapo NS, Maity A (2011). Degradation of insecticides used for indoor spraying in malaria control and possible solutions. Malar J.

[23] Hadaway AB, Barlow F (1963). The toxicity of some organophosphorus compounds to adult *Anopheles stephensi*. Bull World Health Organ.

[24] Bordas E, Downs WG, Navarro L (1953). Inactivation of DDT deposits on mud surfaces. Bull World Health Organ.

[25] Bami HL (1961). Sorption of 75% DDT water-dispersible powder on different mud surfaces. Bull World Health Organ.

[26] Smith A, Hocking KS (1962). Assessment of the residual toxicity to *Anopheles gambiae* of the organophosphorus insecticides Malathion and Baytex. Bull World Health Organ.

[27] Cullen JR, De Zulueta J (1964). Observations on the effect of residual insecticides in experimental huts in Masaka District, Uganda. Bull World Health Organ.

[28] Smith A, Park PO, Hocking KS (1964). Assessment of the kill of *Anopheles gambiae* by the fumigant insecticide Dichlorvos in experimental huts. Bull World Health Organ.

[29] World Health Organization. Test procedures for insecticide resistance monitoring in malaria vectors, bio-efficacy and persistence of insecticides on treated surfaces. WHO/CDS/CPC/MAL/98.12. 1998.

[30] Abbott WS (1925). A method of computing the effectiveness of insecticide. J Econ Entomol.

[31] Mosqueira B, Duchon S, Chandre F, Hougard JM, Carnevale P, Mas-Coma S (2010). Efficacy of insecticide paint against insecticide susceptible and resistant mosquitoes - Part 1: laboratory evaluation. Malar J.

[32] Van Tiell N (1961). Field performance of dieldrin/resin wettable powders on sorptive mud surface. Bull World Health Organ.

[33] Gerolt P (1961). Investigation into the problem of insecticide sorption by soils. Bull World Health Organ.

[34] Hadaway AB, Barlow F (1963). The residual action of two organophosphorus compounds and a carbamate on dried muds. Bull World Health Organ.

[35] Bertagna P (1959). Residual insecticides and the problem of sorption. Bull World Health Organ.

[36] Hadaway AB, Barlow F (1964). A note on the sorption of insecticides on tropical soils. Bull World Health Organ.

[37] Gahan JB, Wilson HG, Smith CN (1966). Cheesecloth impregnated with baygon for control of *Anopheles quadrimaculatus* Say. Bull World Health Organ.

[38] Hadaway AB, Barlow F. Assessment of two possible pre-treatment methods of preventing sorption of insecticide residues by Dried Mud. WHO/MAL/462. 1964. PMC2555266 14315727

[39] Chanda E, Chanda J, Kandyata A, Phiri FN, Muzia L, Haque U (2013). Efficacy of Actellic 300 CS, pirimiphos methyl, for indoor residual spraying in areas of high vector resistance to pyrethroids and carbamates in Zambia. J Med Entomol.

[40] Oxborough RM, Kitau J, Jones R, Feston E, Matowo J, Mosha F (2014). Long-lasting control of *Anopheles arabiensis* by a single spray application of micro-encapsulated pirimiphos-methyl (Actellic300 CS). Malar J.

[41] Rowland M, Boko P, Odjo A, Asidi A, Akogbeto M, N’Guessan R (2013). A new long-lasting indoor residual formulation of the organophosphate insecticide pirimiphos methyl for prolonged control of pyrethroid-resistant mosquitoes: an experimental hut trial in Benin. PLoS ONE.

